# Effect of social media use on food safety risk perception through risk characteristics: Exploring a moderated mediation model among people with different levels of science literacy

**DOI:** 10.3389/fpsyg.2022.963863

**Published:** 2022-09-30

**Authors:** Jie Zhang, Hsi-Chen Wu, Liang Chen, Youzhen Su

**Affiliations:** ^1^Center for Internet and Governance Research of Sun Yat-sen University, Guangzhou, China; ^2^School of Journalism and Communication, Tsinghua University, Beijing, China; ^3^Department of Communication Arts and Sciences, The Pennsylvania State University, State College, PA, United States

**Keywords:** social media use, risk characteristics, food safety risk, risk perception, science literacy

## Abstract

Food safety risk (FSR) is becoming a vital issue for public health, and improving public awareness of FSR through social media is necessary. This study aims to explore specific mechanisms of FSR perception; it first categorizes 19 risk characteristics into two variables, dread and efficacy, and then examines how social media use affects perceived FSR through both variables. Additionally, the study explores the moderating effects of source credibility and science literacy on the mechanisms of FSR perception. Based on a nationwide online survey (*N* = 2,015) of more than six salient food safety issues in China, the study found that exposure to food safety risk information on social media can help improve perceived FSR based on the proposed “dread–efficacy processing model” (DEPM), where dread stimulates perceived risk, while efficacy suppresses risk perception. Moreover, source credibility intensifies the effect of social media use on efficacy appraisal, whereas science literacy exerts a “double-weakening” influence on dread appraisal. Theoretical and practical implications of the findings are discussed.

## Introduction

Food safety risk (FSR), described as “the presence of physical, chemical, or biological contaminants that are unexpected or unidentified on the product label” (Nardi et al., 2020), has become a critical issue for public health. Contaminated food, such as infant formula in China tainted with melamine (Gossner et al., 2009) and heavy metals in food, has been proved to be harmful. Additionally, controversial products such as genetically modified food (Ho et al., 2020) and trench or gutter oil, called *di-gou-you* in Chinese (Wu et al., 2014), may potentially cause harm. These proven or potential harms should be the subject of research by experts, and the appropriate government agencies must respond to all relevant societal consequences. Laypeople, however, might not have access to firsthand FSR information, and thus, this information gap between laypeople and experts or government officials may result in unnecessary divergence and distrust. This calls for the news media to function as a bridge to reduce distrust and enhance mutual understanding. Communication scholars, therefore, often start with media attention or use by laypeople (see Yang et al., 2016; Ho et al., 2020), assuming that media attention and use represent important resources whereby the general public can obtain firsthand information. We also assume that psychological processing is motivated by external stimuli such as FSR information intake resulting from media use. Thus, exploring the relationship between external and internal factors is important. Regardless of the social functions of media or underlying psychological assumptions, media use is a valuable factor that may influence perceived FSR and the related psychological mechanisms.

A further question concerns why social media rather than other types of media use is a focus regarding FSR. With the emerging role of the mobile internet, the public can freely obtain FSR information online and communicate FSR concerns *via* social media such as Weibo and WeChat (Magistad, 2012), partially bypassing mass media censorship (Mou and Lin, 2014). As a result, more than 1.1 billion Chinese online users (71.6% of the total Chinese population) spend nearly 26.9 h per week online (China Internet Network Information Center [CNNIC], 2021). Clearly, social media has already become the main source of information. Therefore, to raise the FSR perception of the general public, investigating how social media use (SMU) affects public risk perception is necessary.

A large body of empirical evidence supports the idea that SMU affects individuals’ perceived risk in various domains (Yang et al., 2016; Ali et al., 2019; Oh et al., 2021; Zhu et al., 2021). Nonetheless, how this use affects the public’s perceived risk regarding certain topics remains unclear. Some scholars have partially examined this question using a heuristic-systematic processing model (Yang et al., 2016) or based on a cognitive-emotional framework (Oh et al., 2015; Oh et al., 2021), but most of their studies have focused on individual cognition without considering risk itself or its characteristics. This study, by contrast, aimed to consider the characteristics of FSR as mediators for explaining how SMU affects FSR perception. Two theoretical assumptions are that how individuals perceive certain risks depends on (1) specific risk domains and (2) heterogeneous characteristics of risk. Specifically, this study first compared and modified two widely used risk characteristic frameworks from Slovic and Sandman, respectively (Slovic et al., 1980; Sandman et al., 1993; Covello and Sandman, 2001; Vassie et al., 2005), ensuring that they fit for the food safety domain. Then, we integrated the two risk characteristic frameworks into one to explain how SMU affects FSR perception.

Unlike superstitions or rumors with definitive “right or wrong” properties, the nature of risk is a kind of perceived uncertainty about potential loss or harm. Perceived uncertainty is involved in each step of the risk perception mechanism–from obtaining risk information (i.e., SMU) to risk information processing (i.e., risk characteristics framework) to risk perception. Such uncertainty makes it difficult for the general public to process FSR information according to media content alone. As a result, laypeople take source credibility or trustworthiness as a complementary cue for believing certain messages or disregarding them as not credible (O’Keef, 1990; Westerman et al., 2014; Yang et al., 2016); this serves as a peripheral route for reducing cognitive load. Yet how source credibility might influence FSR information processing remains unknown, and thus, the moderating effects of source credibility on the influence of SMU are examined in this study.

Lastly, a large body of evidence suggests a spillover effect of science literacy on information processing and decision-making. For instance, people with higher levels of science literacy perform better at eliminating superstition (Turiman et al., 2012), recognizing misinformation (Chou et al., 2018), and debunking health rumors (He et al., 2021). These findings indicate that it may be impossible for people with different literacy levels to perceive risk information in the same way. If the differences in information processing features among a segmented audience (i.e., people with low vs. high science literacy) can be assessed, then customized persuasive campaigns in regard to risk issues could be efficiently implemented for the public’s well-being. In FSR perception, however, little is known about the spillover effects of science literacy; hence, the current study further explored the spillover effect of science literacy on information processing and the risk perception mechanism.

## Materials and methods

### The main effect of social media use on perceived food safety risk

Before defining perceived FSR, it is more essential to understand what is perceived risk first. With the potential harms surrounded, perceived risk is “a combination of the probability, or frequency, of occurrence of a defined hazard and the magnitude of the consequences of the occurrence” (Pidgeon et al., 1992). Such definition consists of three core elements: the first is the potentially harmful environment in a specific situation (sources); the second is the probability of that harms happen to people (possibility); and the third is the severity of such harms (severity). Thus, perceived risk cannot represent the actual risk but an individual’s perceptions of the uncertainty and the possible negative consequences of a specific event or behavior (Jacobs and Worthley, 1999).

Following such logic, perceived FSR has also been discussed by many scholars. As early as in 1997, FSR was generally described as “a function of the probability of an adverse health effect, and the severity of that effect, consequential to a hazard(s) in food” (SCF, 2001), which encompassed both possibility and severity of the risk. Tonsor et al. (2009) considered perceived FSR as “what the individual believes would be the amount of health risk.” With specific risk sources considered afterward, Manning and Soon (2013) defined FSR as a “biological, chemical, or physical agent in food, or condition of food, with the potential to cause adverse health effects.” Further emphasizing the unexpectedness of FSR, Nardi et al. (2020) lately pointed out that FSR–“the presence of physical, chemical, or biological contaminants”–is “unidentified on the product label” so that FSR is a highly hidden health harm to the general public. Taken together all these definitions in the current study, perceived FSR refers to the perceived severity of harms after consuming contaminated or controversial food and the perceived possibility of suffering that harms. Notably, this study expands the range of food risk sources from previously microbiological (i.e., microbe in food), chemical (i.e., heavy metal residuals in food, food additive), and technological (i.e., genetically modified food) three general categories (Yeung and Morris, 2001) into four categories, with the “lifestyle-related” (i.e., high-calorie food; “*di-gou-you*” in food) taken into account. Thus, we totally covered six food safety issues.

Moving onto the relationship between SMU and perceived FSR, it has been found that SMU can positively influence public risk perception. A recent study showed that consumers can quickly acquire accurate information on food by using social media, reducing the risk of food poisoning (Zhu et al., 2021). Likewise, Yang et al. (2016) found that risk perception of food safety could be increased by social media attention. The reason why social media could exert such a positive influence on public risk perception partly lies in its high interactivity, great accessibility, and user-generated content mode (Haas and Wearden, 2003; O’Reilly and Battelle, 2009). Thus, people can quickly obtain various online information and conveniently share it with others (Jang and Baek, 2019), bypassing strict censorship that makes it more difficult to inform the Chinese public food risk information in the mass media age (Mou and Lin, 2014). Thus, perceived FSR can be affected by SMU–a behavioral pattern including users’ exposure frequency to risk information and involvement in sharing information with others (Ali et al., 2019; Zhu et al., 2021). We propose the following hypothesis:

*H1: Social media use is positively associated with perceived FSR.*

### Perceived risk characteristics as predictors of perceived risk

Generally, the magnitude of perceived risk is determined by the probability that risk occurs and its potential loss (Jacobs and Worthley, 1999). In practical, however, the general public’s risk perception about “occurring probability” and “potential loss” of a certain risk is not tantamount to the actual situations for the reason that social or psychological factors would distort public risk perception (Covello and Sandman, 2001). Considering such differences between public risk perception and the actual risk, scholars from the psychometric paradigm regarded perceived risk characteristics like dread, knowledge, and controllability as multidimensional antecedents of perceived risk on a particular hazard (Slovic et al., 1980; Slovic, 1987). Likewise, Covello and Sandman (2001) defined perceived risk characteristics as social/psychological factors that “affect how we judge the actual magnitude of a risk.”

Within the psychometric paradigm, a number of scholars have made efforts in categorizing a variety of risk characteristics through factor analysis. In an earlier study, Slovic (1987) labeled the first category of risk characteristics as “dread risk” where the ratings of perceived lack of control, dread potential, and fatal consequences were highly intercorrelated, and the second category was named “unknown risk” in which perceived newness, perceived scientific knowledge, and delay of effects showed high correlation with the second principal component. Compared with Slovic’s (1987) classification, a later extended study (Vassie et al., 2005) made partial modifications: (1) the first category was still “dread,” but the original 3 items were expanded into 12 items; (2) the second category was labeled as “familiarity” where observability and “unknown to those exposed” were added up.

Some scholars, however, considered the aforementioned classifications as neither clear nor representative of the individual risk characteristics because they only derive from researchers’ interpretations from the factor loadings (Oh et al., 2015). Oh et al. (2015) categorized the risk characteristics into two dimensions grounded on a cognitive-emotional framework. On the one hand, they operationalized the cognitive dimension of risk characteristics by knowledge (people’s perception of how well they know a risk), familiarity (people perceive unfamiliar hazards to be risky), and controllability (people perceive controllable risks to be less serious). On the other hand, the emotional dimension was operationalized by dread and immediacy–dread refers to feelings of fright, and immediacy is defined as “to what extent is the risk immediate or likely to occur at a later time” (Fischhoff et al., 1978).

Nonetheless, the limits of all the aforementioned categorizations are unneglectable. First, specific types of risk domains sometimes determine the grouping outcome of risk characteristics (Brun, 1992; Dohle et al., 2010). In a study on the Norwegian public risk perception of the Chernobyl nuclear reactor accident, immediacy was grouped with “dread” belonging to the “cognitive dimension” (Teigen et al., 1988), whereas Oh et al. (2015) considered immediacy as an item of “emotional dimension” based on H1N1 influenza in South Korea. Thus, when it comes to a different context like FSR, the current study has to examine the validity of categorization.

Second, a group of alternative constructs, “outrage factors” proposed by Sandman (1993), was not assigned enough importance in former studies. Outrage factors, defined as impulses predisposing an individual to react emotionally (Covello and Sandman, 2001), not only include nearly all the risk characteristics items, such as voluntariness, controllability, familiarity, from psychometric paradigm or cognitive-emotional framework, but also include many new constructs such as victim identity (i.e., how imaginable a risk is), trust (i.e., people’s trust to relevant individuals or organizations), and moral/ethical nature (i.e., how morally wrong an event is). These new constructs, however, may be vital in predicting perceived risk. Take the “trust” as an example: the Chinese government frequently plays a critical role in handling public issues–a confident and decisive government may moderate Chinese laypeople’s perceived risk in food safety issues; in other words, trust degree to the government may be negatively associated with public perceived FSR in China.

Hence, due to the lack of consensus among different classification frameworks (Slovic et al., 1980; Siegrist et al., 2005; Oh et al., 2015) and enough attention to new constructs of outrage factors (Covello and Sandman, 2001), the current study would first compare and modify two sets of mutually complementary scales from psychometric paradigm (Fischhoff et al., 1978; Vassie et al., 2005) and outrage factors (Sandman et al., 1993; Covello and Sandman, 2001) and then categorize risk characteristics. Considering both psychometric paradigm and outrage factors, we propose our first research question.

*RQ1: What categories do risk characteristics include?*

### Potential mediating effect of perceived risk characteristics

Locating appropriate risk characteristic(s) shown in RQ1 is the first step for probing the underlying risk perception mechanism. After that, it is possible to explore how SMU affects perceived FSR. Under the cognitive-emotional framework, Oh et al. (2015) took cognitive dimension (i.e., knowledge, controllability, and familiarity) and emotional dimension (i.e., dread and immediacy) of risk characteristics as two mediators in risk perception process: they found that emotional dimension of risk characteristics significantly mediates the effect of entertainment media exposure regarding health information on personal/societal-level perceived risk. Because of such result, Oh et al. (2021) further focused on emotional dimension of risk characteristics, considering fear and anger as the mediators of risk perception mechanism to explain how social media exposure affects the public risk perception about 2015 MERS-CoV. They found that social media exposure is first positively related to both emotions, fear and anger, and then in turn affects public’s perceived risk positively. Similarly, this study would also take risk characteristic(s), first extracted in RQ1, as potential mediator(s) to explore the effect of social media usage on perceived FSR. Therefore, we hypothesize that:

*H2: Social media use can affect perceived FSR through perceived risk characteristic(s). (SMU → Risk Characteristic → FSR).*

### Moderating effect of source credibility on risk information processing

Perceived source credibility has been defined as “judgments made by a perceiver…concerning the believability of a communicator” (O’Keef, 1990) which can impact the selective information consumption of users (Xu, 2013; Lin et al., 2016) and eventually affect media effects. The ground of source credibility in this study mainly lies in the diversity of content producers in social media rather than traditional items such as expertise, trustworthiness, and goodwill (Westerman et al., 2014). The main reason for such a switch is that the user-generated content model of social media enables the general public to access a large amount of non-official information (Yang et al., 2016) and users could easily share information. Accordingly, the public may not only rely on organizational sources like WeChat public accounts verified as government/companies but also simultaneously rely on personalized sources like WeChat family groups and friends discover. Thus, assessing people’s trust in multi-sources in social media may be more representative of source credibility in the internet age.

As the risk information seeking and processing (RISP) model suggests, relevant channel beliefs, including trustworthiness and usefulness, could influence information processing (Griffin et al., 1999) especially when relevant information like FSR is highly related to individual health (Yang et al., 2016). For instance, Yang et al. (2016) found that source credibility can positively predict the systematic processing of information. That being said, on the condition that the credibility of FSR information sources is relatively high, people are more likely to have higher perceived FSR or in risk characteristics processing, with the rise of SMU. Thus, we hypothesized that source credibility may intensify the effect of SMU on perceived FSR or perceived risk characteristics.

H3a: Source credibility intensifies the effect of SMU on perceived FSR. (SC*SMU + *→ FSR).*

H3b: Source credibility intensifies the effect of social media use on risk characteristic(s). (SC*SMU + *→ Risk Characteristics).*

### Spillover effect of science literacy on food safety risk perception mechanism

Science literacy refers to “a broad understanding of the methods of science and a general knowledge of some of its specific content” (Fasce and Picó, 2019), concentrating on the technical knowledge of science (He et al., 2021). FSR as a sort of uncertainty perception, science literacy functions as a necessarily exogenous tool to reduce such uncertainty, by indirectly influencing how people process (i.e., perceive risk characteristics) and then perceive FSR information. Many empirical studies exhibited that the higher science literacy an individual has, the less likely for he or she to believe superstition (Turiman et al., 2012), misinformation (Chou et al., 2018), unwarranted information (Fasce and Picó, 2019), and rumors (He et al., 2021) in social media. These results suggested that science literacy may be conducive to risk information processing and FSR perception in the same way because scientists believe that science literacy helps people make appropriate health decisions (Shen, 1975) in a “rational manner.”

**FIGURE 1:**
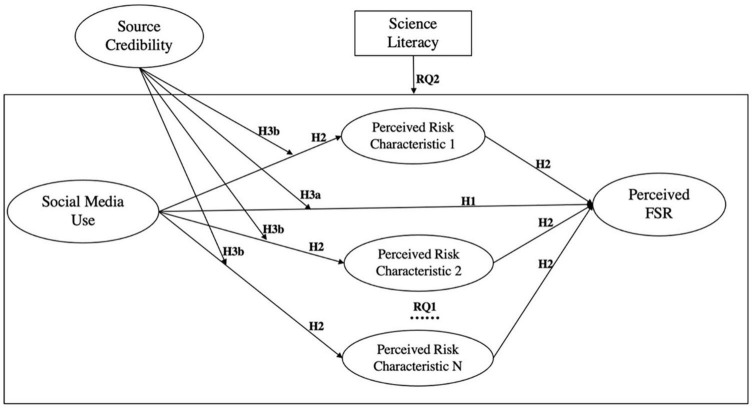
Conceptual model.

The actual risk information processing (risk characteristics) or FSR perception, however, is far more than the issues that could be simply tackled in a “rational manner.” When it comes to FSR perception within the psychometric paradigm, for example, people with higher science literacy may feel less “dread” because they would have more confidence in controlling FSR, making them less cautious in recognizing safe food. After all, high science literacy does not necessarily mean knowing all aspects of food safety, and blind confidence may increase the probability of making the wrong decision. Likewise, in terms of risk information processing, people with higher science literacy may invest more time in collecting information when confronted with “unknown risk” so that they would be less likely to suffer from relevant food issues; but once faced with “familiar risk,” high science literacies may be also overconfident and selectively omit necessary risk information. In short, we need to ask (1) how science literacy interacts with social media use to influence risk characteristic(s) during information processing progress and (2) how science literacy interplays with risk characteristic(s) further to affect FSR perception. Therefore, we proposed RQ2 to examine the spillover effect of science literacy on the whole FSR perception mechanism. A conceptual model including all research questions and hypothesis are exhibited in Figure 1.

*RQ2: Among the people with different levels of science literacy (SL), do the effects of (1)* SMU and (2) perceived risk characteristics on perceived FSR differ? (SL*SMU → FSR; SL*SMU → Risk Characteristics; SL*Risk Characteristics → FSR).

### Sample

From 4 September to 20 September 2017, we applied quota sampling to conduct a nationwide online investigation by “Wen Juan Bao,”^1^ a real-name registration online platform in the Chinese mainland. According to the population proportions of each 31 provinces in the Chinese mainland, we proportionately distributed 2,330 questionnaires across all over 31 provinces and 2015 of them were valid.

Within the valid questionnaires (*N* = 2,015), men accounted for 54.4% and women took up 45.6%. In terms of age, there were 1,122 samples (55.7%) ranging from 18 to 30, 708 (35.1%) from 31 to 45, and 133 (6.6%) from 46 to 60; plus, 31 samples (1.5%) were under 18 while 21 (1.0%) were over 60. When it comes to household income per month, 72.5% (1,461) samples’ incomes varied from 3,001 CNY/month to 15,000 CNY/month, 10.6% (213) had over 15,000 CNY/month and 16.9% (341) had less than 3,000 CNY/month. In total, 1,056 samples (52.4%) were from cities/towns while 959 (47.6%) came from countryside. Moreover, 955 samples (47.4%) had kids under 12 and the other 1,060 (52.6%) did not.

### Measures

#### Perceived food safety risk

Perceived FSR was assessed based on a four-item scale adapted from Oh et al. (2015). The four items measured two personal-level risk perceptions (i.e., “Genetically modified food is harmful to my health” for severity of harms and “It is likely that I would be affected by genetically modified food” for possibility of getting harmed) and two societal-level risk perceptions (i.e., “Genetically modified food causes severe societal harm” and “Genetically modified food is harmful to others”). Each item was measured with a seven-point Likert scale from 1 = “strongly disagree” to 7 = “strongly agree.”

Notably, all four items mentioned above were measured across six important food safety issues in China, including: (1) microbe in food (*M* = 4.59, SD = 1.11, Cronbach’s α = 0.868), (2) heavy metal residuals in food (*M* = 5.00, SD = 1.06, Cronbach’s α = 0.861), (3) food additive (*M* = 4.77, SD = 1.10, Cronbach’s α = 0.878), (4) genetically modified food (*M* = 4.64, SD = 1.13, Cronbach’s α = 0.877), (5) high-calorie food (*M* = 4.65, SD = 1.07, Cronbach’s α = 0.852), and (6) “*di-gou-you*”– trench oil used in food (*M* = 5.27, SD = 1.11, Cronbach’s α = 0.875). We finally calculated the arithmetic average of the six issues’ risk perception mean (*M* = 4.82, SD = 0.84, Cronbach’s α = 0.861), which was subsequently used as the indicator of perceived FSR.

#### Social media use

Social media use was measured with a scale adapted from Shih (2016), who used to measure the Facebook usage of *Pansci* (a science communication website in Taiwan) fans (Shih, 2016). Compared with Shih’s (2016) seven items, we adapted them to four items according to the features of WeChat or Weibo. The four items included reading frequency (e.g., “How often do you read the information about FSR in WeChat or Weibo?”), caring degree (e.g., “How often do you care about the food safety information in WeChat or Weibo?”), sharing frequency (e.g., “How often do you share…?”), and comment frequency (e.g., “How often do you comment…?”). All items were measured with a five-point Likert scale (1 = “Never” and 5 = “Always”). We averaged four items as the indicator (*M* = 3.59, SD = 0.79, Cronbach’s α = 0.858) of SMU.

#### Perceived risk characteristics

We used a 19-item scale adapted from Fischhoff et al. (1978), Slovic et al. (1980), Sandman et al. (1993) and Covello and Sandman (2001). Specifically, seven items (ethical/moral nature, human or natural origin, victim identity, fairness, trust, accident history, and information transparency) were exclusively from Sandman et al. (1993), Covello and Sandman (2001), five items (interest manipulation, the number of people affected, cost to avoid potential risk, personal knowledge, and known to science) were exclusively from Fischhoff et al. (1978), Slovic et al. (1980), and the other seven items (effects on children, dread, delayed effects, voluntariness, catastrophic potential, controllability, and familiarity) were shared by both. Each item was measured with a seven-point Likert scale (1 = “strongly disagree” and 7 = “strongly agree”); for example, item voluntariness was operationalized by “People have to confront with the risk of residual heavy metal in food.” We would average appropriate items as the indicator of perceived risk characteristics.

#### Source credibility

To cover as diverse online sources as possible, six types of social media sources, including organizational/official accounts [(a). news agencies, (b). government, (c). corporates] and personalized/non-official accounts [(d). personal media, (e). friends discover, (f). WeChat Groups], were considered in the six-item scale. For example, “To what extent do you think the food safety information from government WeChat official account is credible?” Six items were measured with seven-point Likert scale ranging from 1 = “very incredible” to 7 = “very credible” (*M* = 4.36, SD = 0.849, Cronbach’s α = 0.826).

#### Science literacy

A six-question true-false test adapted from a previous study (Johnson et al., 2015) was used to measure science literacy. The questions were presented such as “All radioactivity is manmade” (false), “Antibiotics kill viruses as well as bacteria” (false). Samples received one point for each correct answer and zero points for incorrect or “Don’t know” responses, and sum scores range from 0 to 6. We used the sum of six questions’ scores (*M* = 3.93, SD = 1.40) as the indicator of science literacy.

#### Control variables

Demographic features such as sex (0 = female, 1 = male), age [1 = “<18,” 2 = “(18, 30),” 3 = “(31, 45),” 4 = “(46, 60),” 5 = “>60”], household income per month [1 = “≤1,000 CNY,” 2 = “(1001, 3000),” 3 = “(3001, 6000), 4 = “(6001, 9000),” 5 = “(9001, 15000),” 6 = “(15001, 25000),” 7 = “≥25000”], and marriage status (0 = single, 1 = married) are all considered as control variables in this study. Furthermore, another two variables–residential place (1 = in city, 2 = in countryside) and with kids under 12 or not (0 = no, 1 = yes)–are also taken into account for the reasons that: (1) lifestyle difference between city and countryside in mainland China may influence how residents use social media and perceive FSR (e.g., people living in the countryside may experience less food contamination so that they systematically perceive less FSR than people living in the big city do); (2) many people with kids under 12 may be more sensitive to FSR because they feed their children and have the responsibility to make sure that what their children intake is safe.

### Analytical approach

We first applied principal component analysis (PCA) to address RQ1 in SPSS 27 to extract components of perceived risk characteristics, which would be subsequently considered as the mediator(s) in the risk perception mechanism. Next, in multiple linear regression analysis, we used model 63 of the PROCESS v4.0 (Hayes, 2017) in SPSS 27 to examine H1, H2, and H3 and explore RQ2. Moderated mediating effects were tested *via* the *bootstrap* method.

## Results

### The categorization of risk characteristics

As Table 1 shows, the first component was highly correlated with interest manipulation, the number of people affected, and cost to avoid potential risk [exclusively from Fischhoff et al.’ (1978) items]; component 1 was also correlated with ethical/moral nature, human or natural origin, and victim identity [exclusively from Covello and Sandman’ (2001) items]. The rest six items (effects on children, dread, delayed effect, voluntariness, catastrophic potential, and accident history) were included in both Slovic et al.’ (1980) and Covello and Sandman’ (2001) scales. Thus, component 1 consisted of 12 items in total, and it explained 44.68% of the variance.

**TABLE 1:** Principal component analysis (rotated solution) of 19 items.

S19 Items	Component 1	Component 2
Interest manipulation	0.864	0.122
Ethical/moral nature	0.862	0.119
The number of people affected	0.854	0.206
Effects on children	0.845	0.160
Human or natural origin	0.830	0.158
Dread	0.826	0.209
Victim identity	0.795	0.311
Delayed effects	0.789	0.242
Voluntariness	0.785	0.239
Cost to avoid potential risk	0.774	0.241
Catastrophic potential	0.764	0.315
Accident history	0.760	0.383
Personal knowledge	0.064	0.841
Controllability	0.179	0.809
Information transparency	0.163	0.803
Familiarity	0.301	0.788
Trust to government	0.196	0.734
Known to science	0.342	0.709
Fairness	−0.496	−0.383
Variance	44.68%	23.16%
	68%	

Rotation method: Varimax with Kaiser Normalization. Rotation converged in three iterations.

The second component was closely associated with personal knowledge and known to science, which was exclusively from Slovic, and was also highly related to information transparency and trust to government (exclusively from Sandman). Plus, both Slovic’s and Sandman’s scales shared the items’ controllability and familiarity. Together, component 2 has six items, explaining 23.16% of the variance. Therefore, these two components cumulatively explained 68% of the total variance. The single item fairness (exclusively from Sandman) shown in Table 1, however, was negatively correlated with both component 1 and component 2–hence, we eliminated the fairness and did PCA again. After eliminating “fairness,” as Table 2 shows, the first 12 items were also highly correlated with component 1, and the other 6 items were closely associated with component 2. The variance, however, component 1 and component 2 explained 70% of the variance after eliminating “fairness,” slightly higher than before. Therefore, 18 items of the perceived risk characteristics can be grouped into two components.

**TABLE 2:** Principal component analysis (rotated solution) after eliminating “fairness”.

18 Items	Component 1 (dread)	Component 2 (efficacy)
Ethical/moral nature	0.867	0.119
Interest manipulation	0.866	0.123
Effects on children	0.858	0.159
The number of people affected	0.854	0.207
Human or natural origin	0.836	0.158
Dread	0.822	0.212
Victim identity	0.791	0.313
Voluntariness	0.787	0.240
Delayed effects	0.787	0.224
Cost to avoid potential risk	0.767	0.237
Catastrophic potential	0.757	0.240
Accident history	0.753	0.316
Personal knowledge	0.061	0.841
Information transparency	0.165	0.810
Controllability	0.179	0.800
Familiarity	0.307	0.783
Trust to government	0.199	0.743
Known to science	0.351	0.712
Variance	44.19%	25.36%
	70%	

Rotation method: Varimax with Kaiser Normalization. Rotation converged in three iterations.

Moreover, component 1 and component 2 in Table 2 were labeled “dread” and “efficacy,” respectively. For component 1, we used Slovic’s version (see Slovic et al., 1980 and Vassie et al., 2005) of naming “dread,” though our 12 items were partially different from theirs–because we integrated some other items from Covello and Sandman (2001). Within our “dread,” food safety issues causing higher dread would also be perceived with more annoying causes of events (i.e., interest manipulation and human-caused), tougher nature of events (i.e., morally wrong, imaginable victims, involuntary, high cost to avoid, and more accidents in history), and severer consequences of events (i.e., negative effects on children, numerous people affected, high delayed effects, and catastrophic). That being said, our “dread,” in the FSR context, can assess how the general public perceives the cause, nature, and consequence of certain FSR issues as “dread.” Thus, averaged “dread” over six food safety issues (*M* = 4.87, SD = 0.78, Cronbach’s α = 0.962) served as the first risk characteristic of food safety events.

On the other hand, we named component 2 as “efficacy,” deriving from self-efficacy theory, which stated that one’s confidence in their abilities can affect their performance (Bandura and Adams, 1977). However, dealing with FSR is not only to do with individuals, but also with multi-stakeholders consisting of government and scientists, etc.; thus, the second component “efficacy” in this study included both self-efficacy and efficacy to other stakeholders. Specifically, the public would feel more confident in controlling FSR when they have higher self-efficacy (i.e., higher personal knowledge, more familiar with the risk issues, and higher perceived controllability) or efficacy to other stakeholders (i.e., more trust to government/scientists, higher information transparency). Furthermore, efficacy (*M* = 4.33, SD = 0.80, Cronbach’s α = 0.895) in this study also measured the average efficacy over six food safety issues. Taking “dread” and “efficacy” as the two categories of risk characteristics in the FSR context, RQ1 was addressed to some extent.

### Moderated mediation model of risk perception mechanism

As Table 3 exhibits, we further used model 63 of PROCESS v4.0 in SPSS 27 to examine the specific mechanism of FSR perception in different conditions. First of all, the effect of SMU on perceived FSR is significant that social media usage positively influences perceived FSR in this moderated mediation model (β = 0.06, *p* < 0.001), supporting H1. Overall speaking, SMU, dread, efficacy, source credibility, science literacy, and their relevant interactions can jointly explain 79.13% variance of perceived FSR, exhibiting a good fit.

**TABLE 3:** Moderated mediation regression models.

	Dread	Efficacy	Perceived FSR
Intercept	−0.22	−0.17	0.14
Sex (*male* = *1, female* = *0*)	0.09***	0.07*	0.01
Age	0.01	−0.06*	0.00
Marriage status	−0.03	−0.01	−0.05
Household income per month	0.01	0.03*	−0.01
Residential place	0.02	−0.01	0.00
With kids under 12 or not	0.04	0.08	−0.04
Social media use	0.24***	0.19***	0.06***
Source credibility	0.28 ***	0.44***	0.00
Science literacy	0.10***	0.01	−0.07***
Source credibility × social media use	0.02	0.10***	0.03*
Science literacy × social media use			0.03*
Dread			0.89***
Efficacy			−0.08***
Science literacy × dread			−0.03*
Science literacy × efficacy			0.00
*F*-value	52.15***	91.67***	505.38***
Adjusted *R*^2^	22.26%	33.48%	79.13%

All coefficients in Table 4 are standardized coefficients β where *p < 0.05, **p < 0.01, and ***p < 0.001. Sex, age, marriage status, household income per month, and with kids under 12 or not are considered as six control variables.

### Mediating effects of dread and efficacy on risk perception

Secondly, as Table 4 shows, both dread and efficacy can partially mediate the effect of SMU on perceived FSR: on the one hand, SMU positively affects dread (β = 0.24, *p* < 0.001), and then dread strongly promotes perceived FSR (β = 0.89, *p* < 0.001); on the other hand, the higher social media usage becomes, the higher efficacy about the food issues is (β = 0.19, *p* < 0.001), with efficacy subsequently negatively predicting perceived FSR (β = −0.08, *p* < 0.001). Plus, the mediating effect of dread is 82.6% (*p* < 0.001) while the mediating effect of efficacy is −5.6% (*p* < 0.001)–two mediators, dread and efficacy, can jointly explain 77% of the variance in the effect of SMU on perceived FSR. Hence, H2 is attested.

**TABLE 4:** Two risk characteristics as parallel mediators.

	B (SE)	95% LLCI	95% ULCI
***Direct effect of SMU on perceived FSR SMU → FSR***			
Social media use (SMU)	0.06*** (0.01)	0.04	0.09
***Indirect effect of SMU on perceived FSR SMU → Drea → FSR***			
SMU	0.32*** (0.02)	0.28	0.37

*p < 0.05, **p < 0.01, and ***p < 0.001.

#### Moderating role of source credibility

Third, Table 3 indicates that source credibility can generally intensify the positive effect of SMU on perceived FSR (β = 0.03, *p* = 0.013), but such effects mainly exist among the people with relatively higher science literacy according to Table 5, partially supporting H3a. More importantly, based on Table 3, moderating effects of source credibility on specific information processing (risk characteristics) are mixed: it is true that the more people trust certain FSR information sources, the higher positive effect of SMU on efficacy would have (β = 0.10, *p* < 0.001); but the interaction of source credibility and SMU cannot significantly influence dread (β = 0.02, *p* = 0.419). The mixed results suggest that source credibility can only intensify the effect of SMU on promoting efficacy rather than stimulating dread about risk characteristics, partially supporting H3b.

**TABLE 5:** Conditional effects analysis of the moderated mediation model.

IVs	Conditions		B (SE)	95% LLCI	95% ULCI
***Conditional direct effect of stocktickerSMU on perceived stocktickerFSR SMU → FSR***					
SMU	High SL	High SC	0.10*** (0.02)	0.07	0.14
	High SL	Low SC	0.07*** (0.02)	0.03	0.10
	Low SL	High SC	0.04 (0.02)	0.00	0.09
	Low SL	Low SC	0.01 (0.02)	−0.04	0.05
***Conditional indirect effect of SMU on perceived FSR via dread SMU → Dread → FSR***					
SMU	High SL	High SC	0.18*** (0.03)	0.12	0.23
	High SL	Low SC	0.15*** (0.03)	0.10	0.20
	Low SL	High SC	0.38*** (0.05)	0.29	0.47
	Low SL	Low SC	0.35*** (0.05)	0.26	0.44
***Conditional indirect effect of SMU on perceived FSR via efficacy SM → Efficacy → FSR***					
SMU	High SL	High SC	−0.02 (0.01)	−0.03	−0.01
	High SL	Low SC	−0.10 (0.00)	−0.01	0.00
	Low SL	High SC	−0.03 (0.01)	−0.05	−0.01
	Low SL	Low SC	−0.01 (0.01)	−0.03	0.00

*p < 0.05, **p < 0.01, and ***p < 0.001. SMU, social media use; FSR, perceived FSR; SL, science literacy; SC, source credibility.

#### Spillover effects of science literacy

We further explored the spillover effects of science literacy on the whole FSR information processing and perception mechanism. Table 3 shows that the interaction of science literacy and SMU positively predicts perceived FSR (β = 0.03, *p* = 0.02), indicating that the effect of SMU on perceived FSR does differ among people with different levels of science literacy. People with higher science literacy would generally perceive higher FSR than their counterparts with the increasing usage of social media, partially answering RQ2.

**FIGURE 2:**
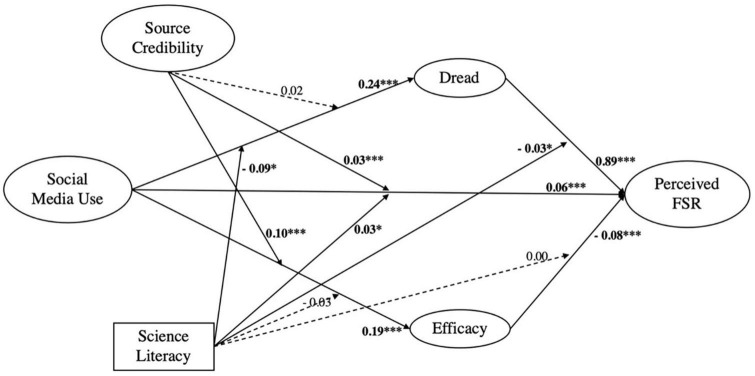
Path coefficients of regression analysis results (*N* = 2,015). *p < 0.05, ***p < 0.001, and all coefficients are standardized beta.

When it comes to specific indirect paths, however, Table 3 suggests that there are no significant differences of efficacy (β = −0.03, *p* = 0.13) and then of perceived FSR (β = 0.00, *p* = 0.77) among people with different levels of science literacy, which are also testified in Table 5 to some extent. Nonetheless, science literacy plays a significant role in another path *via* dread: science literacy can not only negatively moderate the effect of SMU on dread (β = −0.09, *p* < 0.001), but also negatively moderate the effect of dread on perceived FSR of dread on perceived risk (β = −0.03, *p* = 0.03). Such “double-weakening” spillover effects of science literacy can also be observed from Table 5 that with the decrease of science literacy, SMU would exert greater effects on dread, and dread subsequently stimulates perceived risk to a larger extent. Such results suggest that (1) people having higher science literacy feel less dreadful than those having lower science literacy with the increasing usage of social media and that (2) science literacy can dampen the positive effect of dread on perceived FSR among people with different science literacy levels, further answering RQ2. In short, overall path coefficients are shown in Figure 2.

## Discussion

Grounded on six salient food safety issues in China, we first categorized 18 items of risk characteristics into two independent variables, dread and efficacy, and then examined how the two risk characteristics mediate the effects of SMU on perceived FSR, with lastly exploring the moderating effects of source credibility and the spillover effects of science literacy on FSR information processing/perception mechanism.

The first contribution of this study lies in the scale integration for measuring risk characteristics in food safety issues. Following the psychometric paradigm (Fischhoff et al., 1978; Slovic et al., 1980) and outrage factors (Sandman et al., 1993; Covello and Sandman, 2001) under food safety issues, we integrated them into “DEPM” which theoretically contributes to risk information processing. Our 18-item “DEPM” alternatively focuses on people’s subjective appraisals of certain risk issues rather than their cognitive or emotional responses (Oh et al., 2015; Oh et al., 2021). “Dread” in this study represents the extents to which an individual perceives the cause, nature, or consequence of food risk issues as annoying, tough, and severe, respectively, whereas “efficacy” refers to people’s confidence in controlling the potential FSR, and such confidence derives from multi-stakeholders including laypeople themselves, government, and scientists. Such scale integration enriches the risk information processing framework from the perspective of risk features rather than the traditional cognitive-emotional framework (Oh et al., 2015; Oh et al., 2021) or heuristic-systematic model (Yang et al., 2016).

Compared with Oh et al.’s (2015) cognitive-emotional framework and Oh et al.’s (2021) recent “fear–anger” emotion model, our DEPM is a multidimensional framework mainly grounded on people’s assessment of visible risk characteristics. On the one hand, Oh et al.’ (2015) “emotion dimension” consisting of dread–immediacy or fear–anger (2021) was a single-dimensional construct merely involving static feeling outcome, with possible reasons, nature, and impacts of a risk event neglected. By contrast, our 12-item “dread” is a multidimensional construct that is capable of describing the emotion of individuals throughout the whole risk appraisal process from cause (e.g., human or natural origin), nature (e.g., victim identity) to consequences (e.g., effects on children). On the other hand, the “cognitive dimension” of Oh et al. (2015) simply included three items knowledge (people’s perception of how well they know a risk), familiarity (people perceive unfamiliar hazards to be risky), and controllability (people perceive controllable risks to be less serious), essentially belonging to self-efficacy. Besides the three self-efficacy items, our DEPM further considered individuals’ efficacy to others like “trust to government,” “known to science,” and “information transparency”–because actual risk issues are involved with multi-stakeholders. Together, our multidimensional DEPM may be more comprehensive and reliable, especially in the context of food safety issues.

The second contribution manifests in the relationship between media use/information source and risk perception. This is one of the first studies examining the effects of SMU/source credibility on FSR perception through risk characteristics. Not only have we confirmed the direct positive influences of SMU on perceived FSR but also have we attested that both dread and efficacy partially mediate the effect of SMU on risk perception–using more social media to obtain FSR information can promote both dread and efficacy, and then these two risk characteristics can significantly influence perceived risk. Such findings partly echo with Oh et al.’s (2021) results that social media exposure first positively influences two emotions–fear and anger–with these two emotions then positively predicting perceived risk. Compared with Oh et al.’s (2021) results, this study differs in the mediating effects where Oh et al. (2021) only focused on emotion mechanism, whereas our “dread–efficacy” mechanism is grounded on multidimensional risk characteristics.

Plus, we further found that source credibility amplifies the effects of SMU on promoting efficacy other than stimulating dread. A possible explanation for this is the credibility difference among different sources. Our source credibility measured six different online sources in WeChat (i.e., traditional news media, government, corporates, personal media, friends discover, and WeChat groups) in seven-point Likert scale, but people trust government (*M* = 5.01, SD = 1.23) and traditional media (*M* = 4.51, SD = 1.09) most, with giving least trust to personal media (*M* = 4.08, SD = 1.18) and friends discover (*M* = 4.10, SD = 1.20). Such descriptive data suggest that government and traditional media contribute most to the source credibility of participants. Additionally, considering the Chinese government and state-owned traditional news media often regard calming the public down as a priority when confronting food safety issues, such framing strategy makes it difficult for the general public to be sufficiently informed with negative messages about food safety in time. Therefore, four conditions–(1) government and state-owned traditional media contributing most to source credibility, (2) efficacy including trust to government/official organization, (3) dread not involving government, and (4) Chinese government/traditional news agencies’ framing strategy in social media focusing on stressing positive management efficacy to control FSR–together lead to the intensifying impact of source credibility on “SMU → efficacy” rather than on “SMU → dread.” The moderating effects of source credibility confirmed that people do not process risk information solely *via* central route like media content (Cacioppo and Petty, 1984); on the contrary, the attribute of source functions as a peripheral route for people to reduce cognitive loads and make the decision quickly in risk issues (Cacioppo and Petty, 1984).

Thirdly, this is one of the first studies probing the spillover effects of science literacy on risk information processing and perception mechanism. Interestingly, we found that science literacy positively moderates the promoting effects of SMU on perceived FSR in general, but the specific mechanism is a “double-weakening” process: science literacy first dampens the positive effect of SMU on dread and subsequently weakens the positive effect of dread on perceived risk. Such finding suggests that people with higher science literacy do perceive higher food-safety-related risk with the increasing usage of social media, but they do not perceive risk through dread appraisal–high science literacies lastly exhibit higher perceived risk even if under the condition of lower dread. By contrast, people with lower science literacy perceive risk mainly *via* dread assessment, indicating that risk information about the causes, nature, and consequences of food safety issues works better for the low science literacy audiences in promoting risk perception.

Lastly, back to the perceived FSR phenomenon, our definition mainly concentrated on the technical and operational sides with its potential bias omitted. As we specified in the section “The main effect of social media use on perceived FSR,” we defined perceived FSR as the perceived severity of harms after consuming contaminated or controversial food and the perceived possibility of suffering that harms over microbiological, chemical, technological, and lifestyle-related food safety issues. Such “severity–probability” definition is reasonable and measurable indeed, but the fact that individuals tend to pay more attention to the severity or magnitude of potential consequences than the possibility of getting themselves involved (Yeung and Morris, 2001) has been neglected. That being said, during the survey, it could be difficult for individuals to estimate the probability of catching a food harm so that the measures of FSR might actually reflect the degree of severity or the visibility of a particular food issue instead of the comprehensive perception of FSR.

### Implications and limitations

Under the basic skeleton that “FSR information obtaining (i.e., SMU)–FSR information processing (i.e., risk characteristics)–FSR perception,” there are dual theoretical implications out of this study. On the one side, after integrating former measurements of risk characteristics, we categorize the multidimensional risk characteristics into “dread” (i.e., cause, nature, and consequences of risk event) and “efficacy” (i.e., self-efficacy and confidence to multi-stakeholders), exhibiting high reliability and factor loadings at least in six food safety issues. On the other side, more importantly, the two risk characteristics–dread and efficacy–serve as a risk information processing model (named DEPM in this study) in mediating the effect SMU on perceived risk. To be specific, DEPM assumes that the general public would process risk information in an “arousal-control” way because the essence of risk perception is a kind of “uncertainty gaming.” People would assess the cause (i.e., human or natural origin, interest manipulation), nature (i.e., ethical/moral wrong or not), and consequence (i.e., with delayed effects or not) features of an issue as “dread degree” on the one hand and simultaneously assess the self-efficacy (i.e., personal knowledge or controllability) and confidence to others (i.e., trust to government) as “efficacy degree” on the other hand. The former arouses perceived risk while the latter suppresses it–when dread is much higher than efficacy, huge uncertainty remains in mind, pushing up perceived risk lastly. Dread and efficacy are essentially two contrasting forces, explaining how an individual processes risk information and eventually perceives the risk after obtaining or being exposed to risk information in social media.

When it comes to practical implications, spillover effects of science literacy suggest that audience segmentation strategy in social media age could probably be useful: people with lower science literacy are more likely to assess food safety issues as dreadful, with subsequently arousing risk perception to some extent, whereas high science literacies are less likely to rely on dread assessment for perceiving risk, and they might mainly utilize their knowledge or facts-checking skills to perceive certain risk. In this regard, a good way to arouse perceived risk among people with lower science literacy might be emphasizing “dread” characteristics of risk (i.e., human cause, morally wrong nature, or delayed consequences), but the “dread stimulation” may not work in persuading people with high science literacies. By contrast, presenting factual messages, multi-views and competitive framing might be more useful in arousing the risk perception of people with high science literacy.

From macro perspective, we generally interpretate our total conceptual model (see Figure 2) as a mix of “partnership model” and “behavioral insight model” proposed by Kasza et al. (2022). The former features bidirectional communication, considering multi-stakeholders and laypeople actively engaging in obtaining information and even making decision, where SMU (i.e., share/comment risk information) empowers an interactivity-oriented communication while efficacy (i.e., self-efficacy and confidence to government/experts) takes multi-stakeholder into account. The latter one–behavioral insight model–however, manifests in our DEPM and its interactions with science literacy (a kind of prior knowledge level) and source credibility. We believe that there are fundamentally stable modes about “mind” beneath changing behaviors as long as considering enough qualifications. We also testified that individuals do not perceive risk directly based on analyzing external information (i.e., obtaining risk information from SMU) but from underlying psychological mechanism; moreover, prior knowledge such as science literacy can moderate the mediation effects of “dread.” All these finds suggest that final risk perception is not a pure “rationally information-analyzing” mode but a mix of external information intake and internal psychological mechanism processing under certain qualifications (i.e., trust to information source and science literacy).

This study also has some imitations. The first limitation is the sampling method–quota sampling is a non-probability sampling, increasing the possibility of sampling errors. Second, although the current study covered six salient food safety issues in China, increasing the representativeness of our data, we did not check the internal difference among the six issues. After all, the general public cannot perceive six different food safety issues totally in the same way; thus, future study could consider concentrating on one or two issues like gene-modified food or food additive problem. Third, to cover six different social media sources, the “source credibility” of this study was simplified as a single-dimensional concept only involving “trustworthiness,” with another two important dimensions expertise and goodwill (Westerman et al., 2014) omitted–such simplification might weaken the explanatory power of source credibility. More importantly, another flaw of this study lies in the data collection time range. Our data were gathered in 2017, nearly 5 years ago, meaning that we may omit emerging items of risk characteristics resulting from specific situation like COVID-19 and monkeypox. Thus, future studies could combine qualitative, quantitative, and even computational methods to capture the evolving items of risk characteristics, which are essentially complex and multidimensional. The last shortcoming is involved in the content validity of our newly generated scale for measuring risk characteristics driven by PCA. Methodologically speaking, PCA is a dimension-reduction algorithm driven by data rather than human knowledge, so it could not automatically screen out some overlapping or distinct items within the same component, which calls for the expert knowledge to establish the content validity of the two risk characteristics. Therefore, the future study may need 6–10 experts in FSR field rating our 18-items of “dread–efficacy scale,” even though all of our items are from previously used measurements, and both two risk characteristics have already reached high reliability.

## Conclusion

Within the “risk information obtaining–processing–risk perception” skeleton, exposure to FSR information in social media can help improve perceived FSR through the “DEPM.” Our DEPM features an “arousal-control” process where dread stimulates perceived risk *via* causes, nature, and consequences of risk events while efficacy suppresses risk perception by self-efficacy and confidence to other stakeholders. On top of DEPM, source credibility intensifies the effect of SMU on efficacy appraisal whereas science literacy exerts a “double-weakening” influence on dread appraisal.

## Data availability statement

The raw data supporting the conclusions of this article will be made available upon request.

## Ethics statement

The studies involving human participants were reviewed and approved by the Research Committee, Sun Yat-sen University. The patients/participants provided their written informed consent to participate in this study.

## Author contributions

JZ: research idea, questionnaire design, data collection, and writing for research method. H-CW: data analysis and manuscript writing. LC: research design, manuscript editing, and supervision. YS: questionnaire design. All authors contributed to the article and approved the submitted version.

## Footnotes

1
https://survey.surveybaby.com/#/
